# Histone deacetylase inhibitor ITF2357 leads to apoptosis and enhances doxorubicin cytotoxicity in preclinical models of human sarcoma

**DOI:** 10.1038/s41389-018-0026-x

**Published:** 2018-02-23

**Authors:** Marta Di Martile, Marianna Desideri, Maria Grazia Tupone, Simonetta Buglioni, Barbara Antoniani, Carlotta Mastroiorio, Rita Falcioni, Virginia Ferraresi, Nicola Baldini, Roberto Biagini, Michele Milella, Daniela Trisciuoglio, Donatella Del Bufalo

**Affiliations:** 1 Preclinical Models and New Therapeutic Agents Unit, IRCCS Regina Elena National Cancer Institute, Rome, Italy; 2 Pathology Unit, IRCCS Regina Elena National Cancer Institute, Rome, Italy; 3 Cellular Networks and Molecular Therapeutic Targets Unit, IRCCS Regina Elena National Cancer Institute, Rome, Italy; 4 Medical Oncology, IRCCS Regina Elena National Cancer Institute, Rome, Italy; 5 Orthopaedic Pathophysiology and Regenerative Medicine Unit, Istituto Ortopedico Rizzoli, Bologna, Italy; 6 Department of Biomedical and Neuromotor Sciences, University of Bologna, Bologna, Italy; 7 Oncological Orthopedics, IRCCS Regina Elena National Cancer Institute, Rome, Italy; 8 Institute of Molecular Biology and Pathology, National Research Council, Rome, Italy

## Abstract

Sarcomas are rare tumors with generally poor prognosis, for which current therapies have shown limited efficacy. Histone deacetylase inhibitors (HDACi) are emerging anti-tumor agents; however, little is known about their effect in sarcomas. By using established and patient-derived sarcoma cells with different subtypes, we showed that the pan-HDACi, ITF2357, potently inhibited in vitro survival in a p53-independent manner. ITF2357-mediated cell death implied the activation of mitochondrial apoptosis, as attested by induction of pro-apoptotic BH3-only proteins and a caspases-dependent mechanism. ITF2357 also induced autophagy, which protected sarcoma cells from apoptotic cell death. ITF2357 activated forkhead box (FOXO) 1 and 3a transcription factors and their downstream target genes, however, silencing of both FOXO1 and 3a did not protect sarcoma cells against ITF2357-induced apoptosis and upregulated FOXO4 and 6. Notably, ITF2357 synergized with Doxorubicin to induce cell death of established and patient-derived sarcoma cells. Furthermore, combination treatment strongly impaired xenograft tumor growth in vivo, when compared to single treatments, suggesting that combination of ITF2357 with Doxorubicin has the potential to enhance sensitization in different preclinical models of sarcoma. Overall, our study highlights the therapeutic potential of ITF2357, alone or in rational combination therapies, for bone and soft tissue sarcomas management.

## Introduction

Soft tissue and bone sarcomas are rare and aggressive tumors, including >50 histological subtypes with an overall 5-year survival for all stages of about 55%^1, 2^. Available therapeutic options for sarcoma patients with advanced or metastatic tumors are anthracyclines (Doxorubicin or Epirubicin)-based chemotherapy regimens^3–5^. Despite advances in the understanding of the molecular basis of sarcomas, these are still insufficient for the development of new-targeted strategies to potentially improve the management of localized or advanced sarcomas and the dismal prognosis of patients.

Inhibition of histone deacetylases (HDACs), enzymes regulating chromatin topology and gene expression^6^ are considered as new potential therapeutic agents able to reverse aberrant histone acetylation and gene transcription of cancer cells. HDAC inhibitors (HDACi) have been reported as effective anti-cancer molecules alone or in combination in preclinical^7, 8^ and clinical studies^9–11^. In particular, several preclinical studies demonstrated that HDACi alone or in combination therapy constitute novel therapeutics versus different sarcoma histotypes including Ewing^12, 13^, epithelioid^14^, liposarcoma^15^, synovial and rhabdomyosarcomas^16–18^, and osteosarcoma^19–23^. HDACi activities have been mainly due to their effect on cell growth and survival, on apoptosis or autophagy, or cancer stem cells^12,13,15, 24–27^. On the basis of these preclinical evidences, some HDACi against sarcomas have been moving from phase I to phase II clinical trials alone or in combination anthracyclines^9,10, 28–30^.

Here, we wanted to evaluate the effect of a new-generation HDACi, ITF2357 (Givinostat^®^) in human sarcoma cell lines. ITF2357 is a safe and tolerable pan-HDACi with broad anti-inflammatory properties^31, 32^. ITF2357 anti-tumoral activity has been reported in several hematologic^33–35^ and solid tumor^36, 37^models, however, little is known about ITF2357 activity in sarcomas. In the present study, we investigated the molecular and functional effects of ITF2357 in preclinical models of soft tissue and bone sarcomas. We discovered that targeting HDACs by ITF2357 induces a mitochondrial apoptosis in human sarcoma cells. More importantly, ITF2357 enhances in vitro Doxorubicin (Doxo) cytotoxicity in both established and patient-derived sarcoma cells. Furthermore, combination treatment strongly impaired xenografts tumor growth in vivo, when compared to single treatments.

## Results

### ITF2357 reduces in vitro human sarcoma cell growth and induces apoptosis by activating mitochondrial apoptotic pathway

**Fig. 1:**
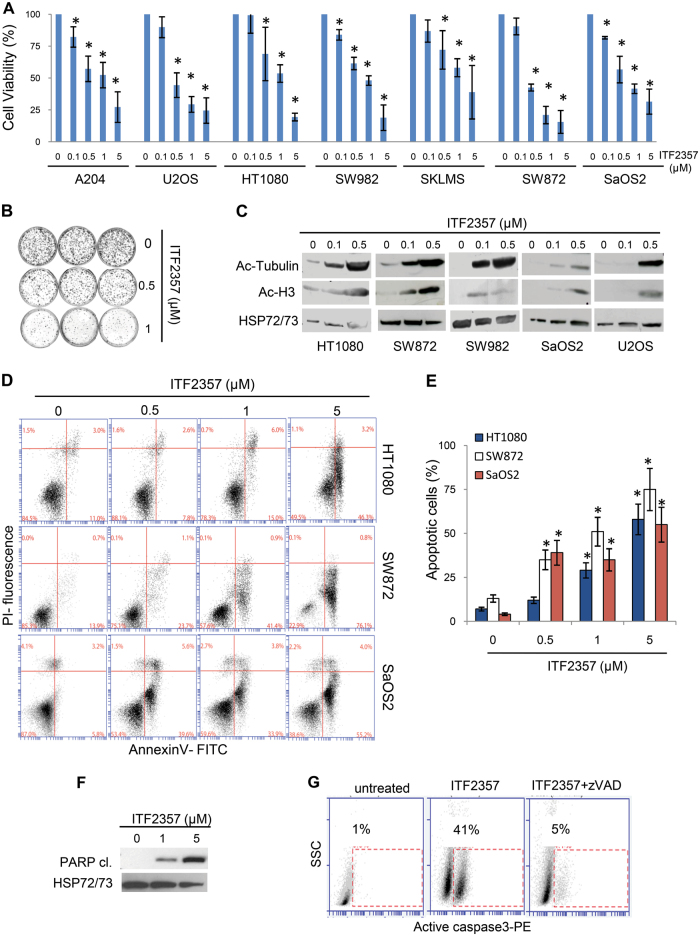
ITF2357 affects survival of human sarcoma cells. **a** Analysis of cell viability by MTT assay in the indicated sarcoma cell lines treated for 72 h with increasing concentrations of ITF2357. The results are reported as “viability of drug-treated cells/viability of control cells” × 100 and represent the mean ± SD of three independent experiments performed in triplicate. **b** Colony formation of HT1080 cells treated for 24 h with increasing concentrations of ITF2357. Representative images are shown. **c** Western blot analysis of acetylated histone H3 (Ac-H3), acetylated α-Tubulin (Ac-Tubulin) in total cell lysates from the indicated cell lines treated with 0.1 and 0.5 μM ITF2357 for 24 h. **d** Representative experiment of apoptotic cells evaluation by AnnexinV/PI staining in the indicated cell lines exposed to increasing concentration of ITF2357 for 72 h. **e** Quantification of apoptosis by AnnexinV/PI staining in the indicated cell lines treated for 72 h with increasing concentrations of ITF2357. The results represent the mean ± SD of three independent experiments. **f** Western blot analysis of cleaved form of PARP (PARP cl.) in total cell lysates from HT1080 cells treated with 1 and 5 μM ITF2357 for 72 h. **c**, **f** HSP72/73 expression was used as loading and transferring control. Western blots representative of two independent experiments with similar results are shown. **g** Flow cytometric analysis of active caspase-3-PE staining in HT1080 cells untreated or treated for 72 h with 1 μM ITF2357 alone or in combination with 50 μM pan-caspase inhibitor zVAD-fmk (zVAD). A representative experiment out of two is shown. **a**, **e** *p* values were calculated between untreated and treated cells, *p < 0.01

Firstly, we assessed the effect of ITF2357 on cell viability of a panel of established human sarcoma cell lines showing different p53 status, including osteosarcoma (SaOS2, U2OS), liposarcoma (SW872), synovial sarcoma (SW982), fibrosarcoma (HT1080), rhabdomyosarcoma (A204), and leiomyosarcoma (SKLMS), that represent the major histological entities of sarcoma family. As shown in Fig. 1a and Fig. 1 suppl., ITF2357 strongly reduced sarcoma cell viability regardless of p53 status, with IC50 values ranging from 3 µM in the p53 null line SaOS2, to 0.57–0.97 µM in p53-mutant lines (SW872, SKLMS) and 0.63–0.88 µM in wild-type (wt) p53 lines (U2OS, SW982, HT1080, A204). To analyze ITF2357 effect on long-term survival of sarcoma cells, we evaluated the clonogenic growth of the representative HT1080 cell line upon ITF2357 treatment. Of note, ITF2357 suppressed colony growth in a dose-dependent manner (Fig. 1b). A 70% reduction in clonogenic ability was observed after treatment with 1 µM ITF2357. Furthermore, exposure to ITF2357 markedly increased histone H3 and α-tubulin protein acetylation in a dose-dependent manner in sarcoma cell lines (Fig. 1c).

By using HT1080, SW872, and SaOS2 representative cell lines, to study in depth molecular mechanisms of ITF2357 we evaluated whether growth inhibition by ITF2357 was due to apoptosis induction. Hence, we assessed the apoptotic effects of ITF2357 by cytofluorimetric analysis of AnnexinV/propidium iodide (PI) staining. A dose-dependent increase in apoptotic cells was observed after treatment with ITF2357 (Fig. 1d, e) in all cell lines even though at different extent. Taken together, these data indicate that ITF2357 treatment inhibited cell proliferation in all sarcoma cell lines tested and that the reduction in the cell viability was associated with induction of apoptosis independently of p53 status.

**Fig. 2:**
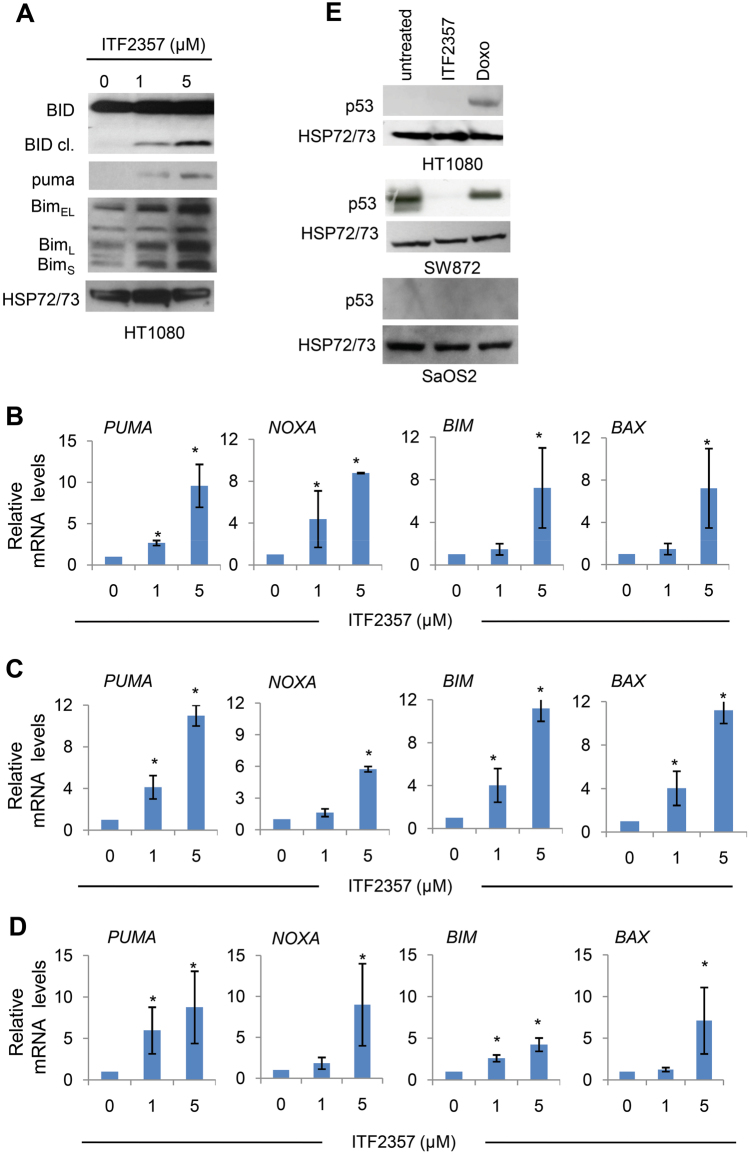
ITF2357 activates mitochondrial apoptosis. **a** Western blot analysis of the indicated BH3-only pro-apoptotic proteins in total cell lysates from the HT1080 cell line treated with 1 and 5 μM ITF2357 for 48 h. **b**–**d** Analysis of the indicated mRNA expression evaluated by qRT-PCR in HT1080 **b**, SW872 **c**, and SaOS2 **d** cell lines exposed to 1 and 5 μM ITF2357 for 48 h. Results are presented as the mean ± SEM of at least two independent experiments. *p* values were calculated between untreated and treated cells, *p < 0.05. **e** Western blot analysis of p53 protein expression in total cell lysates from the indicated cell lines treated for 48 h with ITF2357 (1 μM). As positive control of p53 induction, cells were exposed to Doxorubicin (Doxo, 0.1 μM, 24 h). **a**, **e** HSP72/73 expression was used as loading and transferring control. Western blots representative of two independent experiments with similar results are shown

Focusing our attention on HT1080 cells, we explored whether treatment with ITF2357 induced caspase-dependent apoptosis by using the pan-caspase inhibitor, zVAD-fmk (zVAD). As shown in Fig. 1f, ITF2357 triggered the cleavage of poly(ADP ribose) polymerase (PARP), a typical caspases substrate. We found that about 40% of ITF2357-treated cells were positive for active caspase-3 staining, whereas <5% of zVAD-pretreated cells, as well as, untreated cells were positive for caspase-3 staining (Fig. 1g). In agreement with these results, pharmacological inhibition of caspases protected HT1080 cells against ITF2357-induced loss of cell viability (Fig. 2a suppl.). Next, we analyzed whether ITF2357-induced apoptosis requires the mitochondrial apoptotic pathway. To this aim, we used HT1080 cells overexpressing the anti-apoptotic protein bcl-2. Interestingly, overexpression of bcl-2 counteracted ITF2357-induced cell death, as assessed by cell permeabilization (Fig. 2b suppl.) and AnnexinV surface expression (Fig. 2c suppl.).

Next, we explored in HT1080 cells the expression levels of pro-apoptotic members of the bcl-2 family via western blot (Fig. 2a) and quantitative real-time PCR (qRT-PCR) (Fig. 2b) analyses. Treatment with ITF2357 resulted in a dose-dependent upregulation of Bim isoforms (Bim_EL_, Bim_L_, and Bim_S_). In addition, ITF2357 induced Noxa, Bax, and Puma expression and triggered the cleavage of BH3-only protein Bid (tBid), suggesting tBid recruitment at mitochondria and its involvement in ITF2357-mediated apoptosis. Notably, ITF2357 also transcriptionally upregulated pro-apoptotic BH3-only proteins in SW872 cell line, harboring p53 mutation (Fig. 2c). Of note, we found that ITF2357 transcriptionally upregulated in a dose-dependent manner pro-apoptotic BH3-only genes also in p53 null SaOS2 cells (Fig. 2d), indicating that in our experimental models, transcription of the pro-apoptotic BH3-only genes could involve factor(s) different from p53. Accordingly, ITF2357 induced a decrease of mut-p53 protein levels in SW872 cells, while no accumulation of p53 was observed after ITF2357 treatment of either HT1080 cells, bearing wt-p53, or p53 null SaOS2 cells (Fig. 2e).

Overall, these data indicate that ITF2357 induces apoptosis in a caspase- and bcl-2-dependent manner, through a p53-independent transcriptional mechanism.

### ITF2357 induces autophagy in human sarcoma cells

**Fig. 3:**
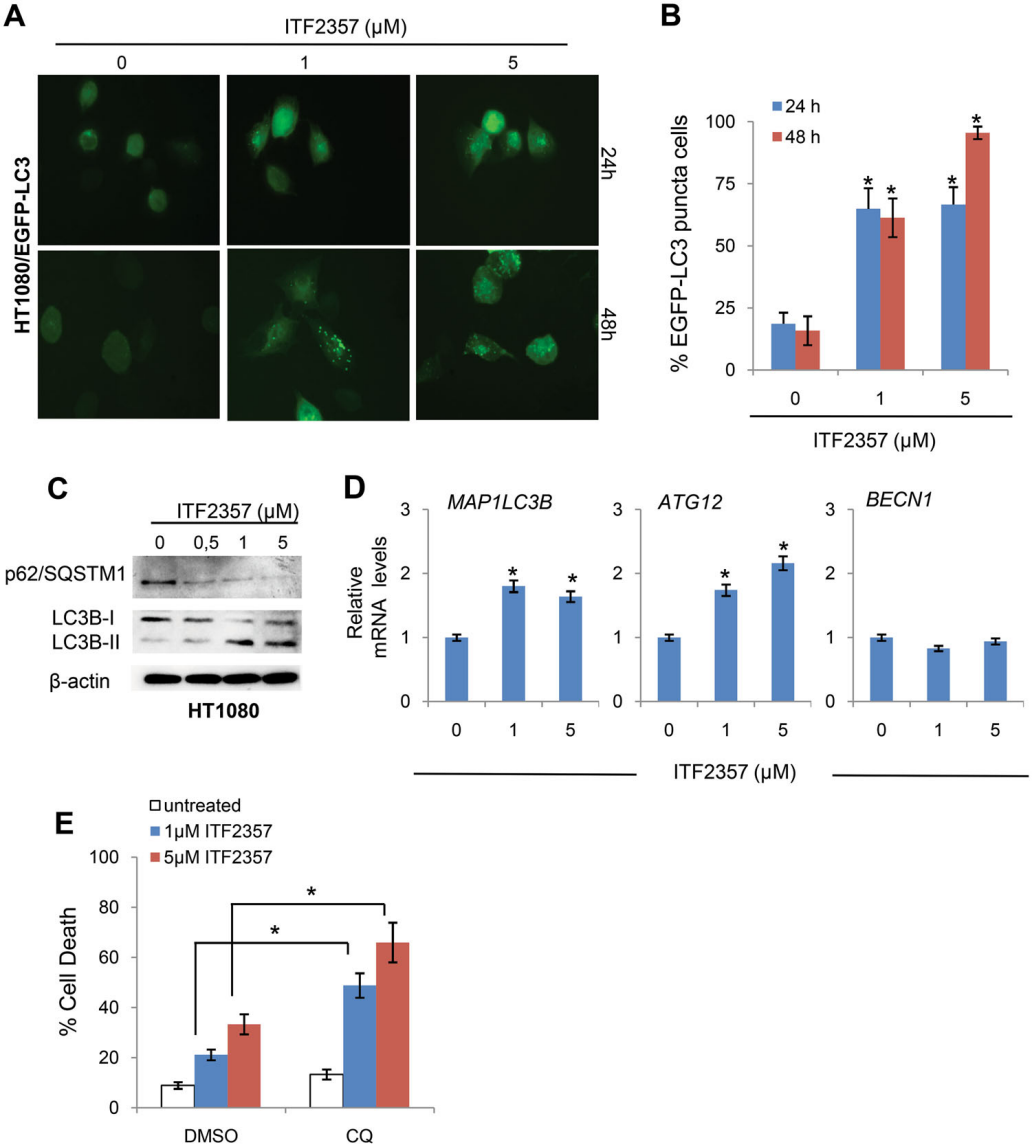
ITF2357 induces a canonical autophagic process. **a** Representative images of autophagosomal structures by fluorescence microscopy in HT1080 cells stably transfected with EGFP-LC3B vector (HT1080/EGFP-LC3) and treated with 1 and 5 μM ITF2357 for the indicated times. **b** Quantification of cells positive for punctate autophagosomal structures treated as indicated in **a**. The results represent the mean ± SEM of three independent experiments. **c** Western blot analysis of microtubule-associated protein 1 (LC3B-I/II) conversion and of p62/SQSTM1 protein expression in total cell lysates from HT1080 cell line treated with increasing concentration of ITF2357 for 24 h. Western blots representative of two independent experiments with similar results are shown. **d** Analysis of the indicated mRNA expression evaluated by qRT-PCR in HT1080 exposed to ITF2357 (1 and 5 μM for 24 h). Results are presented as the mean ± SEM of at least two independent experiments. *p* values were calculated between untreated and treated cells, (*p < 0.05). **e** Analysis of cell death evaluated by PI staining in HT1080 cells exposed to ITF2357 for 48 h in absence or presence of Chloroquine (CQ), a late stage autophagy inhibitor, *p < 0.05. The results represent the mean ± SD of three independent experiments

Because it has been shown that ITF2357 induces autophagy in other cancer models^36, 37^, we investigated the effect of ITF2357 on autophagy also in sarcoma cell lines. In HT1080 cells, the induction of autophagy by ITF2357 was confirmed based on the increase of autophagosomes formation, maturation, and degradation, as well as the increase of lipidated form of LC3B (LC3B-II) and the degradation of p62/SQTSM1, indicating that the autophagic process is progressing to completion. In particular, we evaluated autophagy induction by analyzing LC3B redistribution in HT1080 cells stably expressing the EGFP-LC3B fusion protein (HT1080/EGFP-LC3). As shown in Fig. 3a, b, a time- and dose-dependent enhancement of punctate vesicular structures was observed in ITF2357-treated cells when compared to untreated ones. To further assess the autophagic flux in ITF2357-treated cells, we evaluated the autophagosome maturation in HT1080 cells expressing mRFP-EGFP-LC3 reporter (HT1080/ptf-LC3). Notably, ITF2357 treatment induced the autophagosome-lysosome fusion and complete autophagosome maturation (Fig. 2d suppl.). According with these results, we also found by western blot analysis an increase of LC3B-II form and a decrease of p62/SQTSM1 protein level upon treatment with ITF2357 (Fig. 3c).

Next upon ITF2357 treatment, we evaluated the transcriptional activation of *ATG12*, *BECN1*, and *MAP1LC3B* genes, reported to be induced during the authophagic process^38^. Interestingly, only ATG12 and MAP1LC3B messenger RNA (mRNA) were substantially upregulated in ITF2357-treated cells, while the expression level of BECN1 mRNA was superimposable in untreated and treated cells (Fig. 3d). We also evaluated the biological outcome of autophagy inhibition upon ITF2357 treatment by using chloroquine (CQ), an inhibitor of the lysosomal pH gradient that blocks autophagy at late stage. As shown in Fig. 3e, quantification of cell viability showed an increased cell death when ITF2357 was combined with CQ. Similarly, ITF2357 treatment induced autophagy also in SW872 cells harboring mut-p53, as evidenced by analysis of LC3 puncta formation in SW872/EGFP-LC3B cells (Fig. 2e suppl.). These results indicate that ITF2357 induces a canonical autophagic process both in wt- and mut-p53 sarcoma cells and that ITF2357-induced autophagy shows a cell survival mechanism.

### Activation of Forkhead box (FOXOs) transcription factors has a critical role in ITF2357-induced cell death

**Fig. 4:**
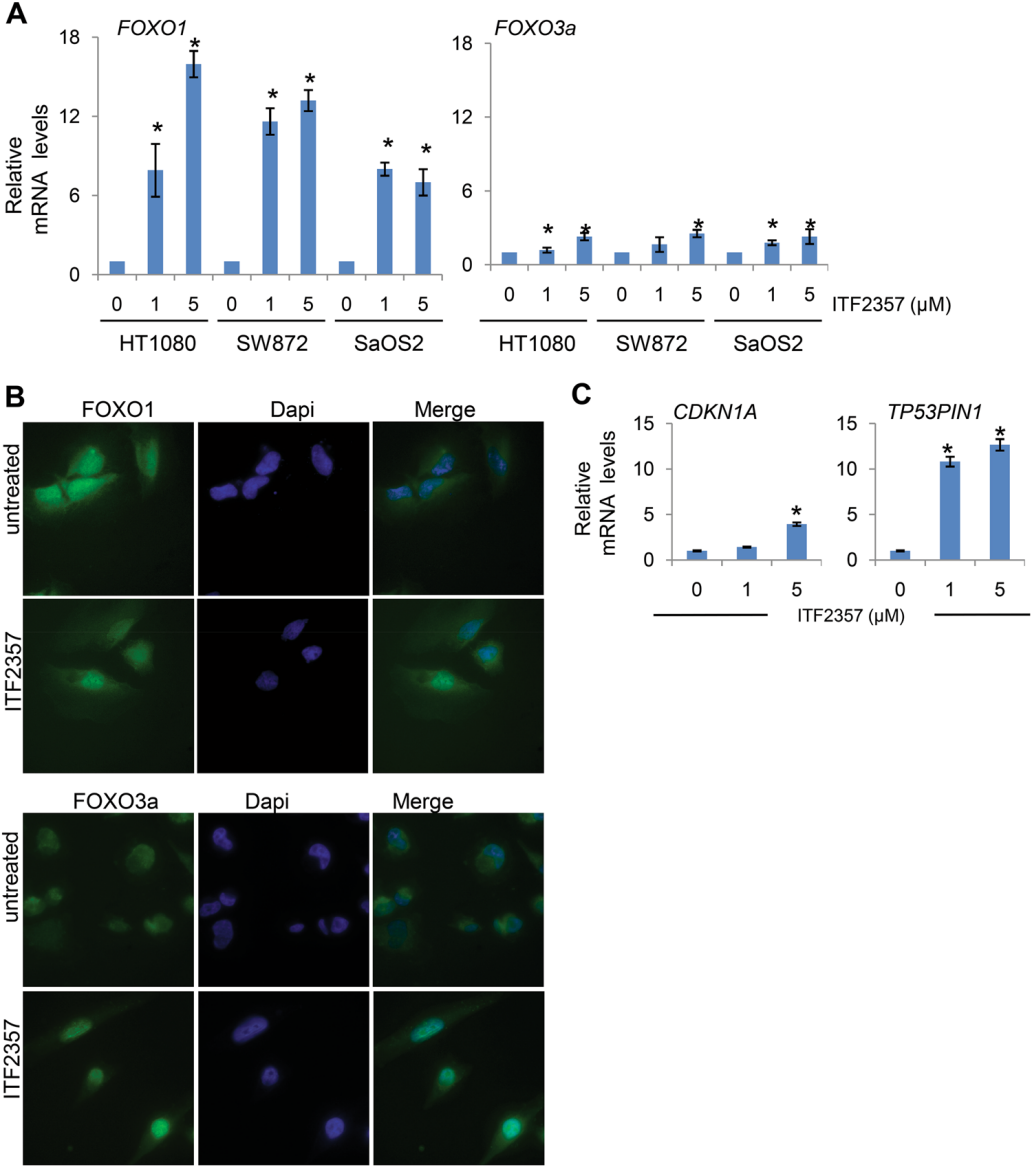
ITF2357 induces FOXOs protein expression and transcriptional activity. **a** Analysis of FOXO1 and FOXO3 mRNA expression evaluated by qRT-PCR in the indicated cell lines treated for 24 h with 1 and 5 μM ITF2357. **b** Representative images of immunofluorescence for FOXO1 and FOXO3 proteins in HT1080 cells after treatment with 1 μM ITF2357. **c** qRT-PCR analysis of the indicated mRNA expression in HT1080 cells treated for 24 h with 1 and 5 μM ITF2357.** a**, **c** Results are presented as the mean ± SEM of two independent experiments. *p* values were calculated between untreated and treated cells, *p < 0.05

**Fig. 5:**
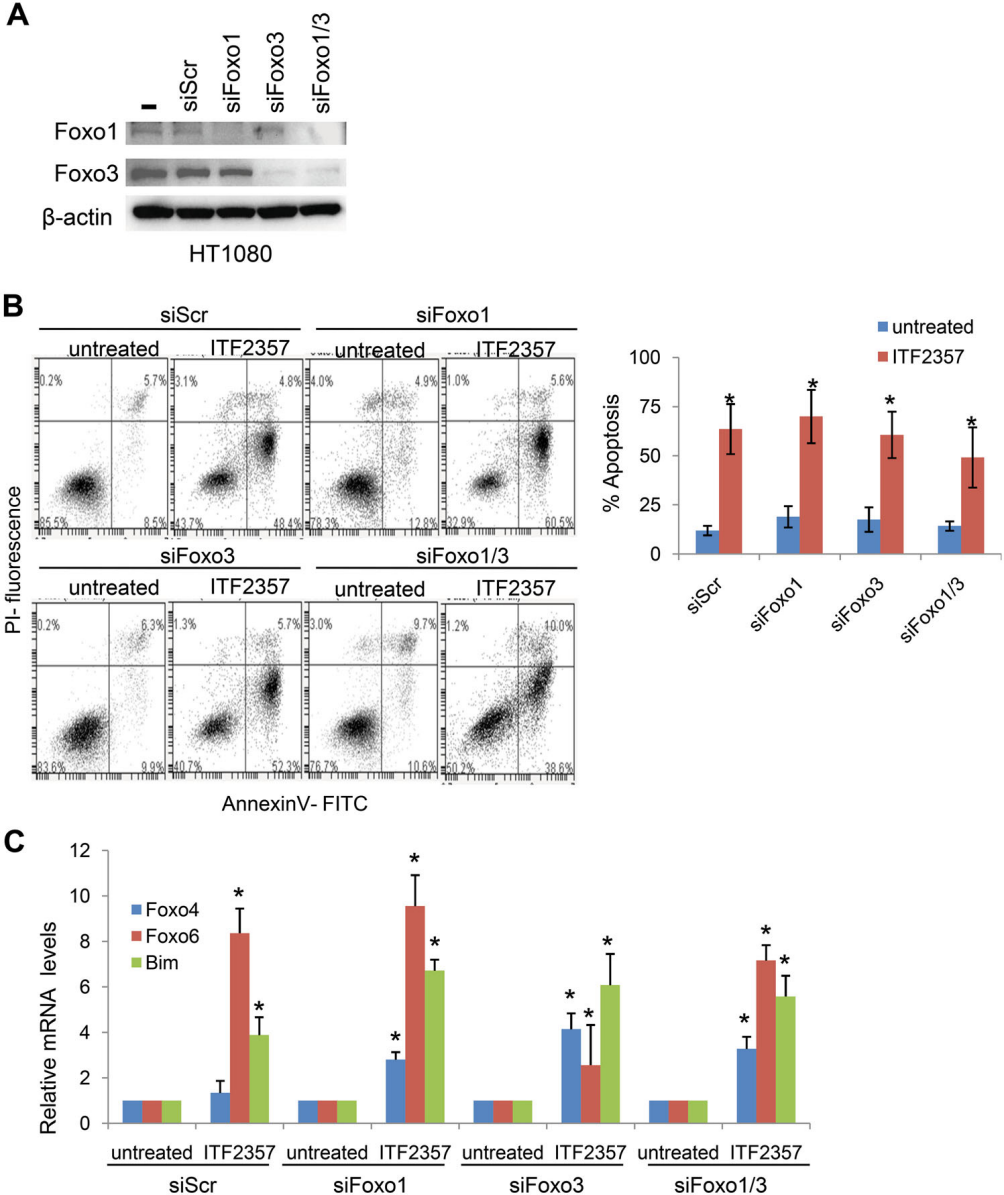
Critical role of FOXOs protein in ITF2357-induced apoptosis. **a** Western blot analysis of FOXO1 and FOXO 3a protein expression in total cell lysates from HT1080 cells after 48 h from trasfection with siRNA-mediated knockdown of FOXO1 and FOXO3a, alone (siFOXO1 and siFOXO3) and in combination (siFOXO1/3) or with a control sequence (siScr). β-actin expression was used as loading and transferring control. Western blots representative of two independent experiments with similar results are shown. **b** Flow cytometric analysis (left panel) and quantification (right panel) of apoptotic cells by AnnexinV/PI staining HT1080 transfected with the indicated siRNAs (siScr, siFOXO1, siFOXO3a, and siFOXO1/3) and exposed to 1 µM ITF2357 for 48 h. The results represent the mean ± SD of three independent experiments. **c** qRT-PCR analysis of the indicated mRNA expression in HT1080 transfected with the indicated siRNAs (siScr, siFOXO1, siFOXO3a, and siFOXO1/3) and exposed to 1 µM ITF2357 for 48 h. *p* values were calculated between untreated and treated cells, *p < 0.05

Several reports suggested a role for FOXO1 and FOXO3a proteins in regulating apoptosis through the expression of BH3-only target genes^39^. Thus, we tested FOXOs expression in three representative sarcoma cell lines exposed to ITF2357. As shown in Fig. 4a, FOXO1 and FOXO3a mRNA levels were increased in three different sarcoma cell lines treated with ITF2357. Next, we analyzed FOXOs nuclear and cytosolic localization in HT1080 cells upon ITF2357 exposure. As reported by immunofluorescence analysis, a similar localization of FOXO1 and FOXO3a was observed in HT1080 cells after ITF2357 treatment (Fig. 4b). In particular, ITF2357-treated cells showed a more evident nuclear localization of FOXO3a in comparison to untreated ones. To further confirm the transcriptional activity of FOXOs, we analyzed the transcription of CDKN1A/p21 and TP53PIN1, two other well-known FOXO-target genes. As reported in Fig. 4c, an increased expression of the two transcripts was observed after ITF2357 treatment. These results suggest that FOXOs might play a role in ITF2357-induced regulation of apoptosis. Thus, we proceeded to confirm the relevance of FOXOs in ITF2357-mediated apoptosis by testing the consequences of siRNA-mediated knockdown of FOXO1 and FOXO3a, alone and in combination (Fig. 5a). Surprisingly, knockdown of FOXO1 or FOXO3a is not associated with a decrease of apoptotic rates in HT1080 cells treated with ITF2357. Notably, similar results were obtained in HT1080 cells after simultaneous knockdown of both FOXO1 and 3a (Fig. 5b). These results were also corroborated by analysis of Bim expression in HT1080 cells upon FOXOs silencing and ITF2357 treatment. Indeed, after FOXOs silencing, Bim expression was still upregulated in response to ITF2357 (Fig. 5c). Interestingly, we found that mRNA of other two FOXO members, FOXO4 and FOXO6, were upregulated in response to ITF2357 when FOXO1 and 3a were downregulated. These results suggest that upregulation of FOXO4 and 6 proteins might sustain ITF2357-mediated apoptosis by upregulation of pro-apoptotic BH3-only protein Bim (Fig. 5c).

### ITF2357 sensitizes human sarcoma cells to doxorubicin treatment both in vitro and in vivo

**Fig. 6:**
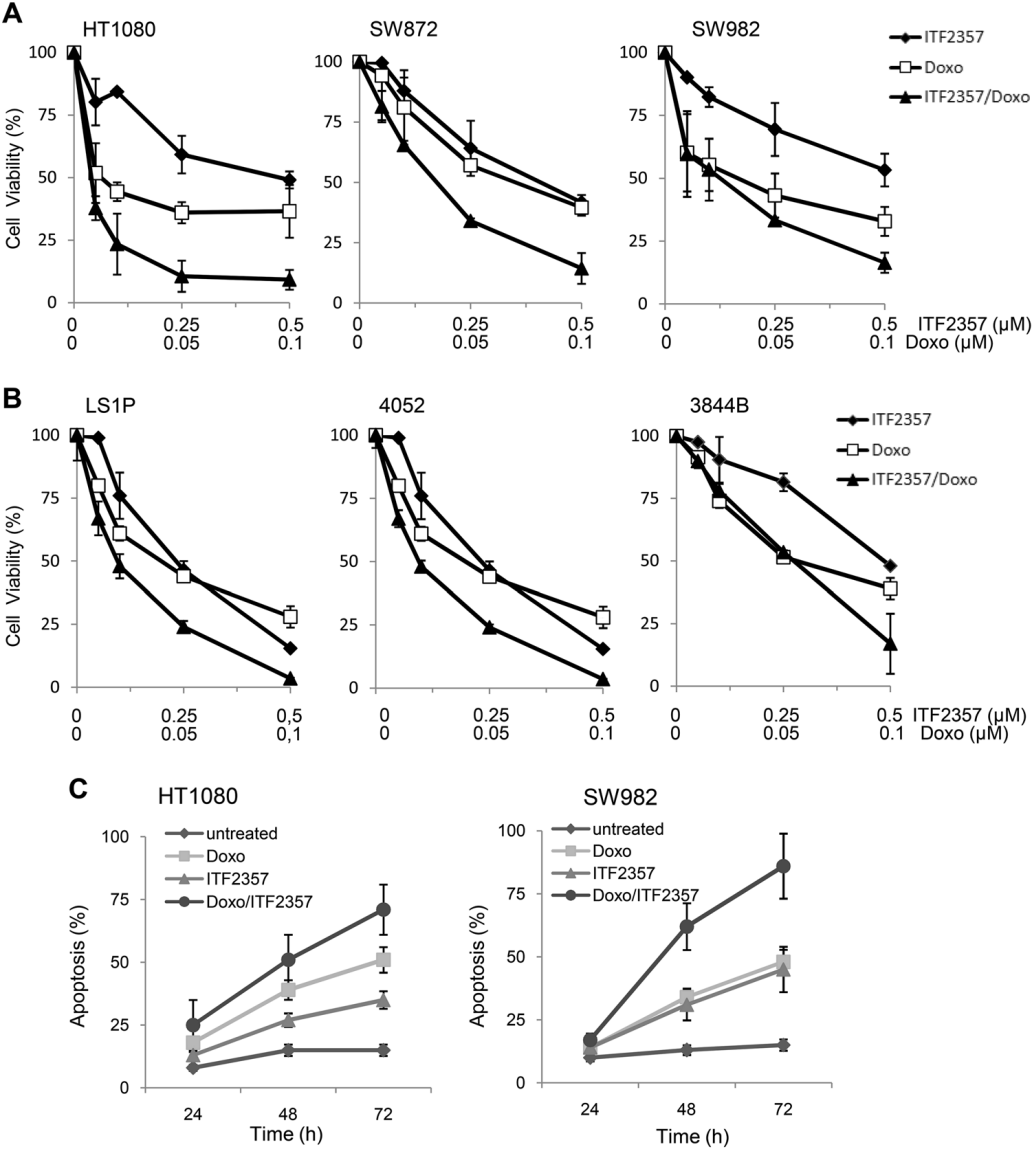
ITF2357 sensitizes human sarcoma cells to Doxorubicin treatment. **a**, **b** Analysis of cell viability by MTT assay in the indicated established **a** and patient-derived **b** cell lines treated with ITF2357 and Doxorubicin (Doxo, drug ratio 5:1) alone or in combination. The results are reported as “viability of drug-treated cells/viability of control cells” × 100 and represent the mean ± SD of three independent experiments performed in triplicate. **c** Cytofluorimetric analysis of apoptotic cells in the indicated cell lines treated with ITF2357 (0.5 μM) and Doxo (0.1 μM) alone or in combination for 24, 48, and 72 h. The results are reported as mean ± SD of three independent experiments

Based on these data, we decided to explore the effect of ITF2357 in combination with conventional sarcoma chemotherapy, such as Doxo, a topoisomerase II inhibitor frequently used as first-line chemotherapeutic for sarcomas^3^. Sarcoma cell lines were exposed to ITF2357 or Doxo alone or in combination (ratio 5:1) and their effect on cell viability was evaluated. Next, combination index (CI) was also calculated by conservative isobologram analysis to evaluate the pharmacological interaction between ITF2357 and Doxo. As shown by growth inhibition curves (Fig. 6a), simultaneous treatment with ITF2357 and Doxo resulted in a higher effect when compared to single treatments, in all cell lines analyzed, although to a different extent. In particular, in HT1080 cells, the combination of ITF2357 (0.5 μM) and Doxo (0.1 μM) caused a growth reduction of about 90% ± 4.7, while single treatments resulted in 50% ± 3 and 63% ± 10 growth reduction after treatment with ITF2357 or Doxo, respectively. A similar effect was also observed in SW872 and SW982 cells. As reported in Table 1, ITF2357 acted in a synergistic manner with Doxo in seven different cell lines. Notably, a significant growth-inhibitory effect of ITF2357 in vitro and a synergistic interaction with Doxo was also evidenced in two-patient-derived sarcoma cell lines (LSP1, 4052), while the effect of the two drugs was additive in 3844B patient-derived sarcoma cell line (Table 1, Fig. 6b). We analyzed modulation of apoptosis following single or combined treatment of two representative cell lines, HT1080 and SW982. In agreement with cell viability data, Annexin V staining shows that all the assessed cell lines have the highest amount of early apoptotic cells (Annexin V+/PI− cells) after combined treatment when compared to the corresponding single treatments (Fig. 6c).

**Table1:** Response to ITF2357 and Doxorubicin in combination of human sarcoma cells lines

		CI values at		
Cell line	Subtype	ED50	ED 75	ED90
HT1080	Fibrosarcoma	0.40	0.04	0.02
SW982	Synovial sarcoma	0.86	0.43	0.30
SW872	Liposarcoma	0.76	0.85	0.96
SaOS2	Osteosarcoma	0.73	0.85	1.04
U2OS	Osteosarcoma	0.64	0.62	1.02
SKLMS	Leiomyosarcoma	0.16	0.09	0.08
A204	Rabdomyosarcoma	0.52	0.51	0.98
3844b	Osteosarcoma	1.08	1.00	0.95
4052	Condrosarcoma	0.98	0.80	0.76
LS1P	Liposarcoma	0.86	0.83	0.84

*CI* combination index, *ED* effective dose

**Fig. 7:**
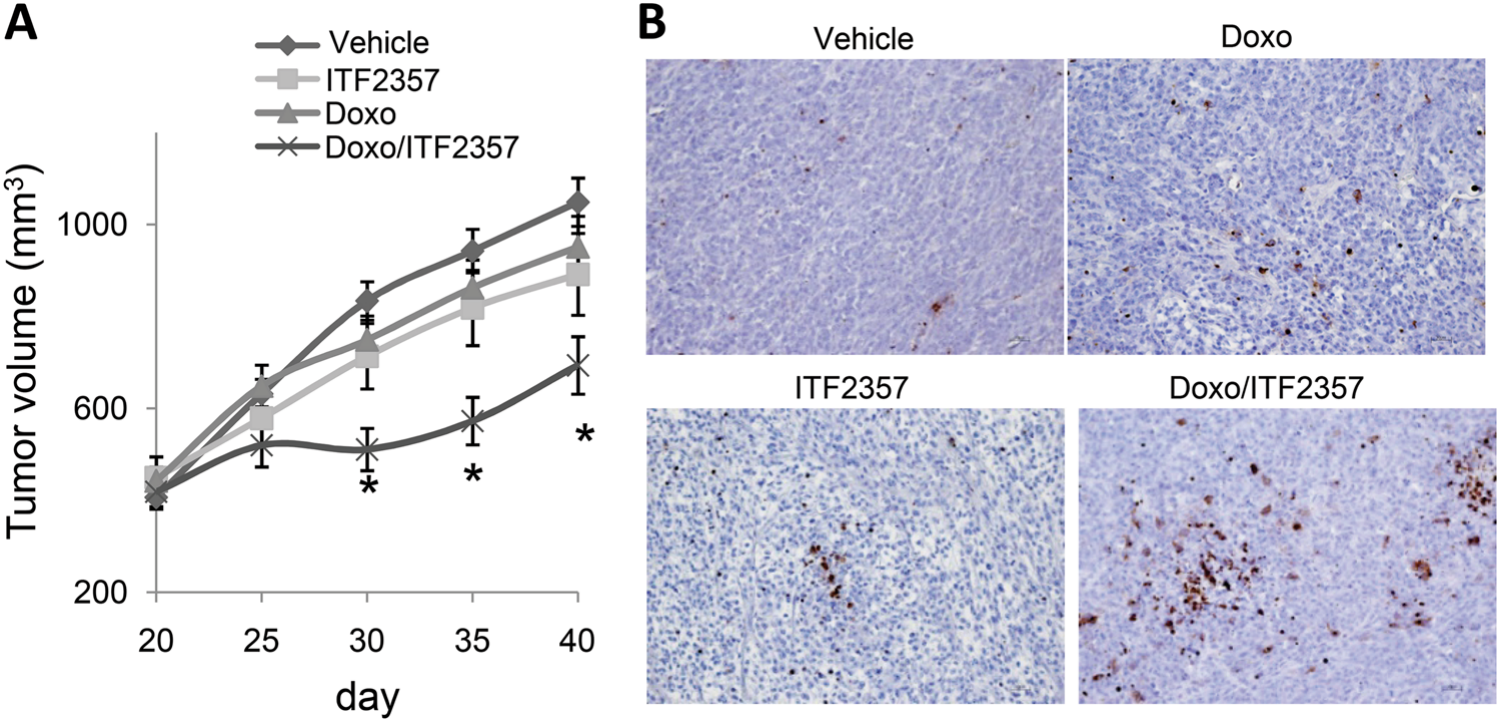
ITF2357 reduces tumor growth and potentiates Doxorubicin effect in vivo. **a** In vivo response of SW982 xenograft to vehicle (daily), Doxorubicin (Doxo, 2.2 mg/kg per week) or ITF2357 (100 mg/kg per day for 4 days) alone, or in combination (weekly Doxo followed by daily ITF2357 for 4 days). Treatments lasted 4 weeks. **b** Representative images of immunohistochemical detection of apoptosis by TUNEL assay of tumor xenografts treated as reported in **a**

The in vitro synergistic cytotoxic activities of Doxo and ITF2357 observed in multiple sarcoma cell lines suggested a therapeutic potential in vivo. To test this hypothesis, we treated established SW982 soft tissue sarcoma^40^ xenografts for 4 weeks with vehicle, Doxo (2.2 mg/kg per week), ITF2357 (100 mg/kg per day for 4 days), or combination Doxo/ITF2357 (weekly Doxo followed by daily ITF2357 for 4 days). As reported in Fig. 7a, treatment with either Doxo or ITF2357 alone did not significantly affect xenografts growth. On the contrary, the combined treatment was significantly inhibitory and was highly tolerated, as no significant weight loss, diet consumption, and postural and behavioral changes were observed. In vivo experiments were also performed in HT1080 xenografts in which treatment with either Doxo or ITF2357 alone slightly affects xenografts growth (about 15% of inhibition). On the contrary, the combined treatment reduces HT1080 tumor growth of about 30% when compared to control xenografts (data not shown). Accordingly, with in vitro data, immunohistochemical analysis of SW982 tumor xenografts showed an increased percentage of apoptotic cells in combination treatments when compared to single ones (Fig. 7b, c). In particular, TUNEL-positive staining nuclei revealed an apoptotic index of 5% for control, 13.6% for Doxo, 13.4% for ITF2357, and 23.6% for the combination. These results indicate that ITF2357 sensitizes sarcoma from different histologic subtypes and p53 mutational status to Doxo both in vitro and in vivo.

## Discussion

Novel therapies are urgent to improve the treatment of sarcomas, a group of different tumors that show a poor prognosis and a low curative outcome^1, 4^.

Using in vitro and in vivo sarcoma models, here we demonstrate that the HDACi, ITF2357, decreases cell viability, activates apoptosis, and increases the growth-inhibitory efficacy of Doxo. We also show evidence about the molecular mechanisms of action of ITF2357-mediated cell death, which implied the activation of mitochondrial pathway of apoptosis, as attested by the increase of pro-apoptotic BH3-only proteins, and both caspase- and bcl-2-dependent cell death. BH3-only proteins are effectors of mitochondrial apoptosis and are able to activate Bax and Bak through direct or indirect mechanisms^41^. In agreement with other groups demonstrating p53-independent transcriptional regulation of some pro-apoptotic BH3-only genes^42^, we found that ITF2357 transcriptionally upregulated, in a dose-dependent manner, Bax, Puma, Noxa, and Bim in cells harboring mutant or wt p53, thus indicating a p53-independent mechanism.

The notion that activation of mitochondrial pathway occurs upstream and before caspases activation is corroborated by results obtained in Bcl-2 overexpressing cells, and by the use of zVAD, a broad-range caspase inhibitor: both bcl-2 overexpression and caspases inhibition strongly reduced apoptosis. Interestingly, we also found that ITF2357 induces a canonical autophagic process, and that inhibition of autophagy strongly increased apoptosis induced by ITF2357, suggesting that in our models, autophagy shows a pro-survival effect. Controversial results have been reported about the effect of autophagy on HDACi-mediated cell death, being observed both cytoprotective and cytotoxic functions of autophagy^43, 44^. We previously reported that ITF2357 in combination with Pemetrexed caused a toxic form of autophagy that subsequently activated a caspase-dependent apoptotic program in lung cancer models^37^. In agreement with other studies, we found that autophagy shows a prosurvival effect. Among them, a recent study shows that FOXO1-dependent pathways are implicated in HDACi-induced autophagy^26^. FOXOs members, include transcription factors with relevant role in gene regulation^39^. They act as tumor suppressor genes and their alterations have been reported in some human cancers^39^, including rhabdomyosarcoma and osteosarcoma^45, 46^. We reported that HDAC inhibition by ITF2357, induced FOXO1/3 upregulation, FOXO proteins nuclear accumulation, and transcriptional activation of FOXO-target genes, such as BH3-only proteins Puma, Noxa, and Bim, thus altering the equlibrium between anti-apoptotic and pro-apoptotic proteins belonging to bcl-2 family, which in turn culminates into mitochondrial alteration, caspases activation, and DNA fragmentation. These data are in agreement with the well-established pro-apoptotic function of FOXO1, through upregulation of Bim or Noxa genes recently described in osteosarcoma^46^. Also the expression of other downstream targets of FOXOs, including CDKN1A/p21 and TP53PIN1, was found to be upregulated in response to ITF2357 in sarcoma models. Knockdown experiments were performed to deeper investigate the role of FOXOs in cytotoxicity mediated by ITF2357. Notably, reduction of both FOXO1 and 3a did not inhibit the induction of Bim expression in response to ITF2357, and consequently did not protect sarcoma cells against ITF2357-induced apoptosis. Since we also observed that in silenced cells, ITF2357 treatment upregulated other two members of FOXO family, FOXO4 and FOXO6, we might hypothesize that FOXO4 and FOXO6 upregulation might sustains ITF2357-mediated apoptosis through upregulation of pro-apoptotic BH3-only protein Bim. Overall, our results evidence similar FOXO-dependent apoptotic mechanisms in response to ITF2357 in sarcoma cells with wt or mutated p53 status.

We also studied the role of ITF2357 in combinatorial regimes with Doxo, the current standard of systemic therapy care for most subtypes of sarcoma. Overall our results demonstrate that (1) co-treatment ITF2357/Doxo strongly inhibits cell viability and determines a significant increase in number of apoptotic cells when compared to single treatments; (2) as evidenced by CI analysis, there is a synergistic interaction of ITF2357 and Doxo to reduce cell viability and induce apoptosis; (3) co-treatment ITF2357/Doxo strongly reduces in vivo tumor growth, whereas monotherapy with either agents exhibit no significant anti-tumor effect; and (4) the clinical relevance of co-treatment ITF2357/Doxo is also evidenced in primary patient-derived sarcoma cells. These evidences demonstrate the potency of this combination therapy in sarcoma models and are in agreement with other sarcoma studies^9,29, 47^.

Taken together, our data indicate that ITF2357 may represent an important potential therapeutic agent against human sarcoma regardless of p53 status, and that the pharmacological combination of ITF2357 with Doxo has the potential to enhance sensitization in different preclinical models of sarcoma. Based on these results, the potential clinical value of ITF2357, over other HDACi agents previously assessed in sarcoma, deserves further studies in the future.

## Materials and methods

### Cell culture and chemicals

Established osteosarcoma (SaOS2, U2OS), liposarcoma (SW872), synovial sarcoma (SW982), rabdomyosarcoma (A204), leiomyosarcoma (SKLMS), and fibrosarcoma (HT1080) cell lines were originally purchased from ATCC (Manassas, VA). Patient-derived osteosarcoma (3844B), liposarcoma (LS1P), and condrosarcoma (4052) cells were obtained from Dr Baldini (Istituto Ortopedico Rizzoli, Bologna, Italy). EGFP-LC3B, ptf-LC3, and GFP-bcl-2 stable clones, obtained as previously described^48^, were cultured in the presence of geneticin (800 μg/ml, Sigma-Aldrich, St. Louis, MO). Cells were routinely tested for mycoplasm presence.

ITF2357 (diethyl-[6-(4-hydroxycarbamoyl-phenyl carbamoyloxymethyl)-naphthalen-2-yl methyl]-ammonium chloride monohydrate) was provided by Italfarmaco (patent WO 97/43251, US 6034096, Milan, Italy). Doxorubicin (Doxo) was obtained from Sigma-Aldrich. Pan-caspase inhibitor zVAD-fmk (zVAD, 50 μM, Sigma-Aldrich) and chloroquine diphosphate (CQ, 25 μM, Sigma-Aldrich) were dissolved in DMSO and water, respectively.

### Analysis of cell viability and apoptosis

The effect of ITF2357 and Doxo on in vitro cell proliferation was assessed following manufacturer’s protocol by measuring 3-[4,5-dimethylthiazol-2-yl]-2,5-diphenyltetrazolium bromide inner salt (MTT, Sigma-Aldrich) dye absorbance. The viability was calculated for each concentration of drugs used as “optical density (OD) of treated cells/OD of control cells × 100.” By using Calcusyn software, we calculated the concentration of drug that reduces 50% of cell viability (IC50). According to pilot experiments, the DMSO concentration (0.05%) used for the experiments did not affect the proliferation of sarcoma cell lines (data not shown). Caspase-3 activation and the presence of apoptotic cells were evaluated in untreated or treated cells staining using active caspase 3-PE antibody (cat. 559565, BD Bioscience, San Jose, CA) and with Annexin V-FITC or AnnexinV-APC and PI staining. Cytofluorimetric analysis was performed by BD C6 Accuri as previously reported^37^.

### Analysis of autophagy

LC3B puncta formation (autophagosomal structures) in EGFP-LC3B-expressing cells, and distribuition/alteration of fluorescent signals (autophagosome maturation) in mRFP-GFP-LC3B-expressing cells were detected by fluorescence microscopy after grown of cells on glass coverslips and fixation formaldehyde (2%, 10 min, room temperature)^38^.

### Western blot and immunofluorescence analyses

Western blot and immunofluorescence analyses were performed as previously described^37, 48^ using the following antibodies: H3 acetylated histone (cat. 06-599), FOXO3 (cat. 04-1007) (Millipore, Billerica, MA), p62/SQSTM1 (cat. sc-28369), p53 (cat. sc-126), Bax (cat. sc-493), Noxa (cat. sc-30209) (Santa Cruz, Biotechnology, Santa Cruz, CA), HSP72/73 (cat. Hsp-01, Calbiochem, San Diego, CA), acetyl-α-tubulin (cat. T7451), Puma (cat. PRS3041)(Sigma-Aldrich), LC3B (cat. 2775S), FOXO1 (cat. 2880) and BID (cat. 20025), Bim (cat. 2819) (Cell Signaling, Danvers, MA). Goat anti-mouse IgG and goat anti-rabbit IgG conjugated to horseradish peroxidase were used as secondary antibodies. Enhanced chemiluminescence was used for detection (Amersham Bioscience, Freiburg, Germany). Representative blots of at least two independent experiments are shown.

### Assessment of synergism between ITF2357 and Doxo

Effects of drugs combination were evaluated using the CI equation through CalcuSyn software (Biosoft, Cambridge, UK). Synergism CI <0.9, additivity 0.9 < CI < 1.1, antagonism CI > 1.1.

### Quantitative RT-PCR analysis

Quantitative real-time PCR analysis was performed to evaluate gene expression, as previously reported^37^. The following primers were used: human Puma forward: 5′-AAGTCAGGACTTGCAGGCGCG-3′; human Puma reverse: 5′-TGGGTCCCAGTCCGTGTGTGT-3′; human Bax forward: 5′-TCCCGGCTCTCTGATCCCCG-3′; human Bax reverse: 5′-GGCTAGGGGAACGCTATATGC-3′; human Noxa forward: 5′-CGCTGACGACGTCCCAGCGTTT-3′; human Noxa reverse: 5′-CGAAGACGGCGTTAT-3′; human Bim forward: 5′-CAGAGATATGGATCGCCCAAG-3′; human Bim reverse: 5′-TGCGTCTGCTACAGTGGTTCTT-3′; human MAP1LC3B forward: 5′-TGGCCCAACCTGTCTGTACTTC-3′; human MAP1LC3B reverse: 5′-AAAAGTACCAGCCGCATGGAG-3′; human ATG12 forward: 5′-GCAGAAAAGGTTAGGCGTTTTG-3′; human ATG12 reverse: 5′-ATGTGCTTGCTCTCCTGGCTA-3′; human BECN1 forward: 5′-AGGAACTCACAGCTCCATTAC-3′; human BECN1 reverse: 5′-AATGGCTCCTCTCCTGAGTT-3′; human FOXO1 forward: 5′-AACCTGGCATTACAGTTGGCC-3′; human FOXO1 reverse: 5′-AAATGCAGGAGGCATGACTACG-3′; human FOXO3a forward: 5′-TGCGTGCCCTACTTCAAGGAT-3′; human FOXO3a reverse: 5′-ACCCGCATGAATCGACTATGC-3′; human TP53PIN1 forward: 5′-GAGGTTGTCACCAACGCACGT-3′; human TP53PIN1 reverse: 5′-TGAGGGAGAGATCCACCTCTG-3′; human CDKN1A forward: 5′-CCTGGCACCTCACCTGCTCTGCTG-3′; human CDKN1A reverse: 5′-GCAGAAGATGTAGAGCGGGCCTTTG-3′; human FOXO4 forward: 5′ACTGTGGCAGGCTTCACTGAAC-3′; human FOXO4 reverse: 5′TCTAGGTCTATGATCGCGGCA-3′; human FOXO6 forward: 5′TCTACGACTGGATGGTCCGT-3′; human FOXO6 reverse: 5′-GGGTCTTCCCTGTCTTTCCG-3′; human GAPDH forward: 5′-TCCCTGAGCTGAACGGGAAG-3′; human GAPDH reverse: 5′-GGAGGAGTGGGTGTCGCTGT-3′; human actin: forward: 5′-ATTGCCGACAGGATGCAGAA-3′; human actin reverse: 5′- GCTGATCCACATCTGCTGGAA-3′.

Each sample was run in triplicate and normalized to GAPDH or actin mRNA to evaluate relative expression. The 2^−ΔΔCt^ method was used to evaluate fold change in gene expression levels, expressed in unit less values.

### In vivo experiments

Procedures relative to animal use and care were authorized and certified by Italian Minister of Health (decree no. 67/97A, protocol 2560/97, Rome Health Service Unit). Regina Elena National Cancer Institute and animal care Unit approved all the procedures involving animals (species, quality and number of animals, discomfort/distress/pain of animals, anesthesia and killing).

SW982 of 5 × 10^6^ or HT1080 cells were intramuscularly injected into immunodeficient athymic mice (6–8 week-old female). Four groups of animals with similar tumor volume were created when the tumors reach the palpability. The following treatments administered for 4 weeks: (1) daily vehicle administration, (2) weekly intraperitoneal treatment with Doxo (2.2 mg/kg), (3) daily oral administration with ITF2357 (100 mg/kg) for 4 days, and (4) sequential Doxo/ITF2357 treatment (weekly Doxo followed by daily ITF2357 for 4 days). Ten animals composed each group. Tumor volume (mm^3^) was daily calculated to evaluate the effect of different treatments on in vivo tumor growth. Animals were also daily monitored for food consumption, body weight, and behavior.

The experiments were repeated twice. At the end of treatment, tumors were excised, fixed in formalin, embedded in paraffin, and stained with hematoxylin and eosin. Tumor inhibition rate (%) was calculated as follows: (mean tumor volume of control group−mean tumor volume of treated group)/mean tumor volume of control group × 100%. The non-parametric Mann–Whitney test was employed to evaluate differences between the groups. Differences were statistically significant when *p* < 0.05.

TUNEL assay (In Situ Cell Death Detection Kit, POD, Roche) was used to detect apoptosis in tumor sections according to the manufacturer’s instructions. Frozen sections of 5 μm (3 for each tumor) were analyzed by light microscopy and apoptotic cells were counted in four high-power fields (×400 magnification).

### Statistical analysis

All experiments were repeated at least two times. Cell viability, apoptosis, and mRNA levels were expressed as means ± SD or means ± SEM, and significances were analyzed with the *t* test. All data were included in the analyses. Based on variation shown in our previous results, we determined the sample sizes by using power analysis^37^. Immunofluorescence and TUNEL analyses were performed by two investigators in blinded and independent manner.

## Electronic supplementary material

supplementary figure legends

Figure 1S

Figure 2S
