# Supplementary material for: Histone deacetylase inhibitor ITF2357 leads to apoptosis and enhances doxorubicin cytotoxicity in preclinical models of human sarcoma
Source: Oncogenesis. 2018 Feb 23;7(2):20. doi: 10.1038/s41389-018-0026-x (PMC5833676; doi:10.1038/s41389-018-0026-x)
Supplement: Supplementary file 1 — supplementary figure legends [file 41389_2018_26_MOESM1_ESM.doc]

SUPPLEMENTARY FIGURE LEGEND

Figure 1S. p53 expression and ITF2357 IC50 values of human sarcoma cell lines. ITF2357 sensitivity expressed as IC50 value and p53 expression evaluated by Western Blot in the indicated human sarcoma cell lines. HSP70/72 expression was used as loading and transferring control. Western blots representative of two independent experiments with similar results are shown.

Figure 2S. HDAC inhibitor ITF2357 induced apoptosis is reduced by Bcl-2 overexpression. (A) Analysis of cell viability in HT1080 cells treated with increasing concentrations of ITF2357 in presence or absence of pan-caspases inhibitor zVAD-fmk (zVAD, 50μM) for 72h. (B) Analysis of cell viability in HT1080 cells transiently transfected with GFP-empty (empty) or GFP-Bcl-2 (Bcl-2) expression vectors and treated for 48 h with increasing concentrations of ITF2357. (C) Quantification of early apoptotic cells (AnnexinV-PE+/PI-) in HT1080 cells transiently transfected with GFP-empty (empty) or GFP-Bcl-2 (Bcl-2) expression vectors and treated for 48 h with increasing concentrations of ITF2357. The results represent the mean ± SD of three independent experiments. (D) Representative images of autophagosomal (yellow) and autophagolysosomas (red) structures by fluorescence microscopy in HT1080 cells stably transfected with ptf-LC3B vector (HT1080/ptf-LC3) and treated with ITF2357 for 24h. (E) Representative images of autophagosomal structures by fluorescence microscopy in SW872 cells stably transfected with EGFP-LC3B vector (SW872/EGFP-LC3) and treated with ITF2357 for 24h .(A,B) The results are reported as "viability of drug-treated cells/viability of control cells" × 100 and represent the mean ± SD of three independent experiments performed in triplicate. (A,B,C) p-values were calculated between untreated and treated cells, *p<0.01.
