# Supplementary figures and images for: Histone deacetylase inhibitor ITF2357 leads to apoptosis and enhances doxorubicin cytotoxicity in preclinical models of human sarcoma
Source: Oncogenesis. 2018 Feb 23;7(2):20. doi: 10.1038/s41389-018-0026-x (PMC5833676; doi:10.1038/s41389-018-0026-x)

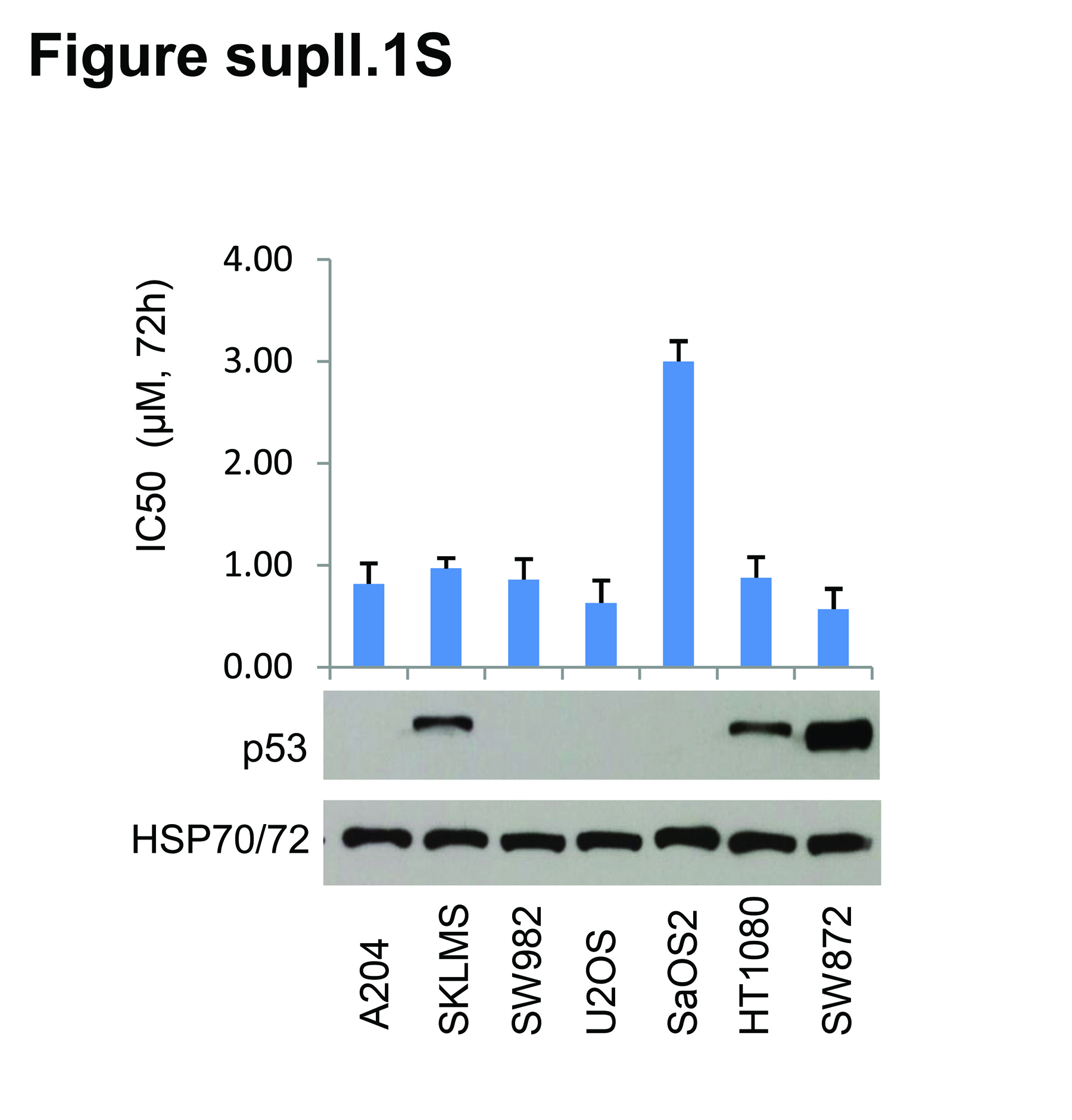

Supplement: Supplementary file 2 — Figure 1S [file 41389_2018_26_MOESM2_ESM.jpg]

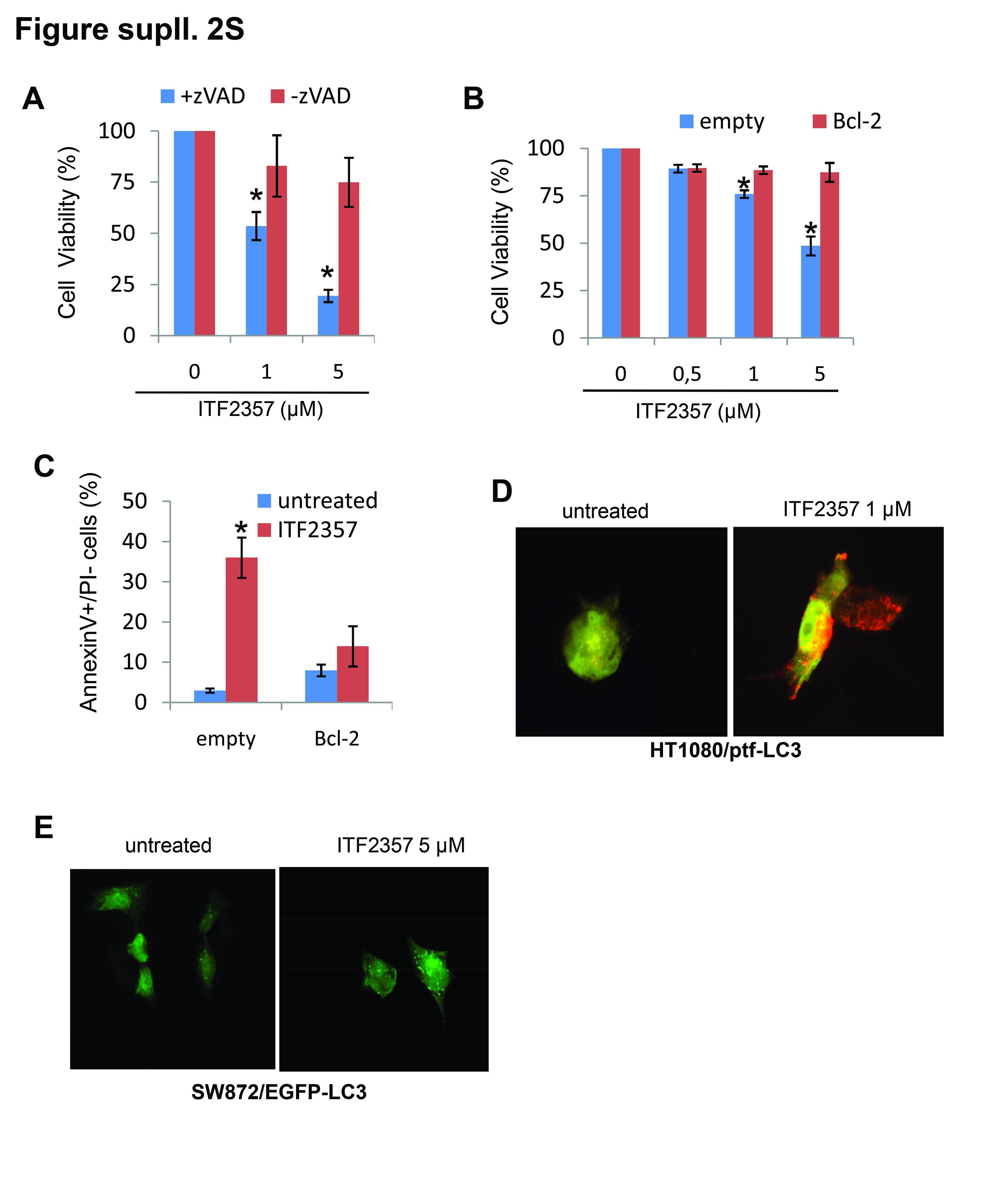

Supplement: Supplementary file 3 — Figure 2S [file 41389_2018_26_MOESM3_ESM.jpg]
